# MiR-30 family prevents uPAR-ITGB3 signaling activation through calcineurin-NFATC pathway to protect podocytes

**DOI:** 10.1038/s41419-019-1625-y

**Published:** 2019-05-24

**Authors:** Yue Lang, Yue Zhao, Chunxia Zheng, Yinghui Lu, Junnan Wu, Xiaodong Zhu, Mingchao Zhang, Fan Yang, Xiaodong Xu, Shaolin Shi, Zhihong Liu

**Affiliations:** National Clinical Research Center of Kidney Diseases, Jinling Hospital, Nanjing University School of Medicine, Nanjing, Jiangsu 210002 China

**Keywords:** Cell signalling, Mechanisms of disease

## Abstract

Urokinase plasminogen activator receptor (uPAR) is upregulated in podocytes of glomerular diseases and crucially mediates podocyte injury through integrin β3 (ITGB3). We previously showed that the miR-30 family maintains podocyte structure and function by inhibiting injurious calcineurin signaling through nuclear factor of activated T cells C (NFATC). Here, we tested whether the miR-30-calcineurin-NFATC and uPAR-ITGB3 pathways, two of the major pathways leading to podocyte injury, could interact. We found that podocyte-specific miR-30 knockdown in mice induced uPAR upregulation and ITGB3 activation, accompanied by proteinuria and podocyte injury. These effects of miR-30 knockdown were reduced using inhibitors of ITGB3, calcineurin, and NFATC, respectively, which are known to be antiproteinuric. These results indicate that miR-30 deficiency leads to calcineurin-NFATC signaling activation, which in turn activates the uPAR-ITGB3 pathway. In cultured podocytes, miR-30 knockdown also activated uPAR-ITGB3 signaling, leading to Rho GTPase activation, synaptopodin downregulation and podocyte injury. To explore uPAR-ITGB3 signaling regulation by miR-30 in podocytopathy development, we treated mice with lipopolysaccharide (LPS) and found that miR-30 was downregulated in podocytes, accompanied by uPAR upregulation and ITGB3 activation. We obtained the same results in cultured podocytes treated with LPS. Podocyte-specific transgenic miR-30 abolished uPAR-ITGB3 signaling and ameliorated podocyte injury and proteinuria in mice. Taken together, these experiments show that uPAR-ITGB3 signaling is negatively regulated by miR-30 through calcineurin-NFATC pathway, a novel mechanism underlying podocyte injury in glomerular diseases. Our study has elucidated the relationship among the crucial players governing podocyte pathophysiology and the antiproteinuric actions of drugs commonly used for podocytopathies.

## Introduction

Podocytes constitute the last layer of glomerular filtration barrier through their foot-processes and slit diaphragms. In recent years, there have been increasing studies showing that podocyte injury can initiate glomerular diseases^1^, and the severity of podocyte injury or loss correlates with that of proteinuria and glomerular injury^2,3^. The mechanisms of podocyte injury are complex and require extensive exploration. Studies have demonstrated that microRNAs are involved in the maintenance of podocyte structure and function, and their dysregulations result in podocyte injury^4^. It has been shown that podocyte-specific Dicer knockout leads to progressive podocyte injury, proteinuria and glomerular sclerosis in mice and that miR-30 family is implicated in the Dicer deficiency-induced podocyte injury^5,6^. More recently, we have shown that miR-30 family members are specifically expressed in podocytes in a glomerulus and that they are downregulated in glomerular diseases, leading to podocyte apoptosis and cytoskeletal damage^7^. Meanwhile, glucocorticoids can restore miR-30 expression, thereby alleviating podocyte injury^7^. We have further demonstrated that miR-30 strongly inhibits calcium/calcineurin-NFATC signaling^8^, which is known to crucially mediate podocyte injury^9–11^. Family members of NFATC (nuclear factor of activated T cells) are substrates of calcineurin, and upon dephosphorylation by calcineurin they are activated and translocated to nuclei where they function as transcription factors to promote target gene transcription^11^.

Urokinase plasminogen activator receptor (uPAR) is a glycosylphosphatidyl inositol (GPI) anchored membrane protein. Upon ligand binding with urokinase plasminogen activator (uPA), uPAR is activated to promote proteolysis of cell adhesion molecules and extracellular matrix, thereby facilitating cell migration^12^. In addition, uPAR can bind to integrins on cell surface and activate intracellular signaling pathways that regulate cell adhesion, migration, proliferation and survival^13–15^. uPAR has a low level of expression under physiological condition and is upregulated during tissue injury, inflammation and remodeling^16^. In kidney, uPAR expression is greatly increased in podocytes in glomerular diseases and experimental podocyte injury models, resulting in cytoskeletal injury through activating ITGB3 signaling and downstream Rho GTPases, CDC42 and RAC1, which are crucial for podocyte normal structure and function^17^.

Since we previously showed that miR-30 family maintains podocyte structure, function and survival, we wondered whether miR-30 could act to prevent uPAR-ITGB3 signaling activation as one of the mechanisms underlying its protective effect. Our present study has demonstrated that miR-30 can inhibit uPAR-ITGB3 signaling through calcineurin-NFATC pathway and that miR-30 downregulation in podocytes leads to the calcineurin-NFATC, and thus uPAR-ITGB3, signaling activation and podocyte injury. We have also shown particularly in animal model of podocytopathy that multiple drugs for glomerular disease treatment can effectively block these pathways, thereby ameliorating podocyte injury.

## Results

### Podocyte-specific miR-30 knockdown activated uPAR-ITGB3 signaling and caused podocyte injury in mice

**Fig. 1:**
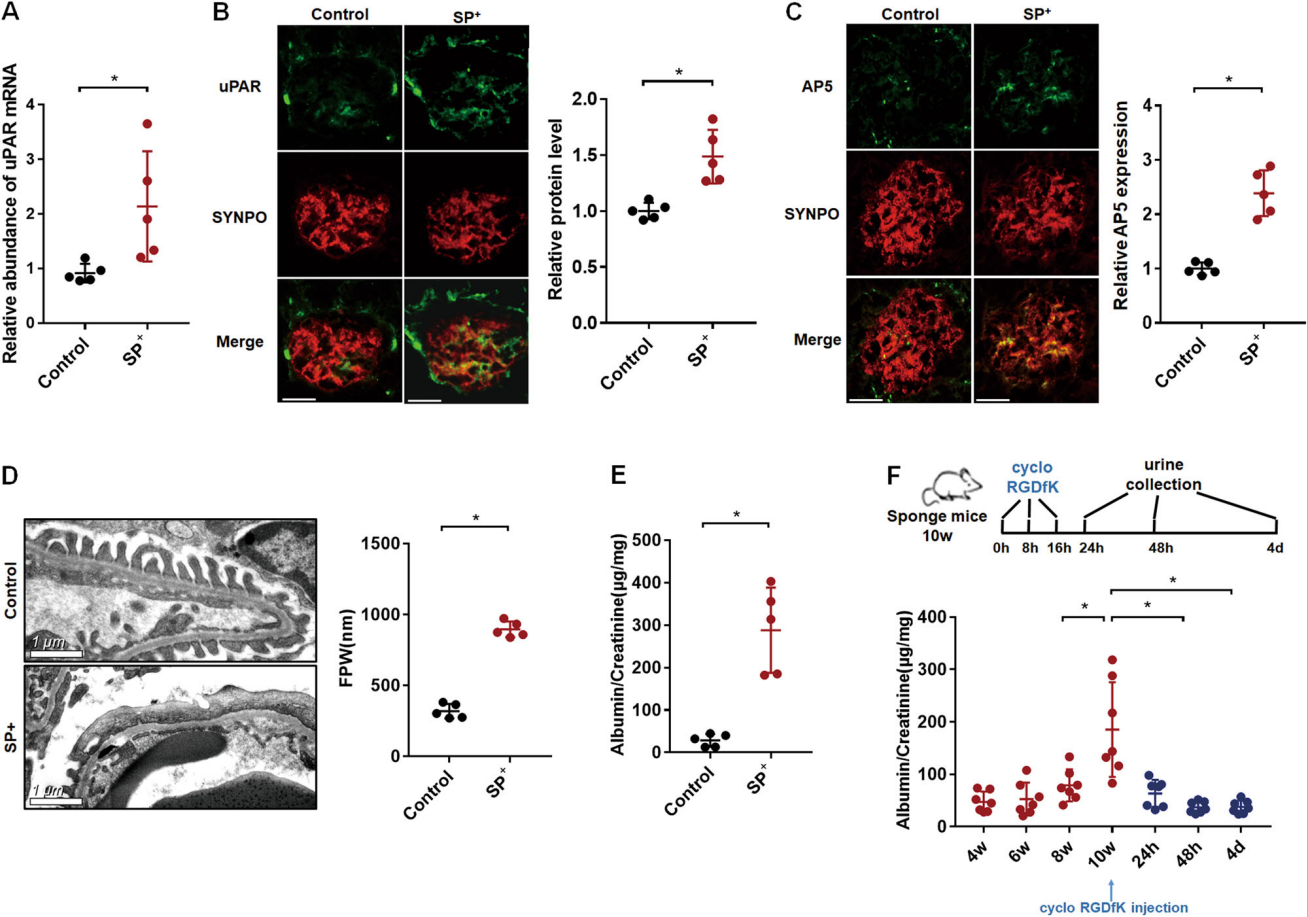
Podocyte-specific transgenic miR-30 sponge activated uPAR-ITGB3 signaling and induced podocyte injury in mice. **a** qRT-PCR analysis of uPAR in isolated glomeruli from the transgenic mice, showing an increase of uPAR mRNA in glomeruli of the SP^+^ mice compared with the control group. **b** IF staining showed that uPAR expression was upregulated in podocytes of the SP^+^ mice, and co-localized with synaptopodin. Magnification in ×600. **c** IF staining using AP5 antibody showed that ITGB3 was activated in podocytes of the SP^+^ mice as evidenced by the colocalization of AP5 staining with synaptopodin. Magnification in ×600. **d** Electron microscopy showed foot process effacement in glomeruli of the SP^+^ mice. **e** ELISA of albumin/creatinine ratio of the urine samples from the SP^+^ mice, indicating the development of albuminuria in the SP^+^ mice; **f** Time course of urinary albumin/creatinine in the SP^+^ mice, showing that albuminuria developed at 10 weeks of age but was abrogated by the ITGB3 inhibitor, cyclo -RGDfK (i.v. 10 mg/kg, 3 injections in 24 h) 24 h after the treatment. The values in the result quantifications were all expressed as the means ± SD. Two-tailed student’s T test was used in (**a**–**e**), while One-way Repeated Measures ANOVA in F, all with *P < 0.05 vs. control group was considered statistically significant. Scale bar = 20 μm. *N* = 5 in both control and SP^+^ groups; *N* = 7 in SP^+^ + cyclo-RGDfK study

We have previously generated and characterized podocyte-specific miR-30 sponge transgenic mice (SP^+^)^8^. In the present study, we used 10 week-old SP^+^ and control mice to examine the effect of miR-30 knockdown on uPAR-ITGB3 signaling in podocytes of the mice. We performed qPCR analysis of uPAR expression in glomeruli isolated from the SP^+^ mice and found an increase of uPAR mRNA compared to control SP^-^ mice (Fig. 1a). Consistently, IF staining showed an increase of uPAR protein in glomeruli of the SP^+^ mice, which was mostly colocalized with a podocyte marker, synaptopodin (Fig. 1b). Meanwhile, ITGB3 was activated prominently in podocytes according to the IF staining using AP5 antibody that recognized active ITGB3 (AP5 positive staining was referred to as AP5 expression) (Fig. 1c). The SP^+^ mice also exhibited podocyte injuries, including foot process effacement (Fig. 1d) and prominent albuminuria (Fig. 1e). To determine the role of ITGB3 activation in podocyte injury of the SP^+^ mice, we treated them with ITGB3 inhibitor, cyclo-RGDfK. SP^+^ mice received 3 injections of the inhibitor in 24 h, and urine samples were collected thereafter at 24 h, 48 h, and 4 days, respectively, for albumin to creatinine ratio (ACR) measurement. The results showed that cyclo-RGDfK significantly decreased ACR at 24 h, and further decreases were observed at 48 h and 4 days, respectively (Fig. 1f). These results indicate that uPAR-ITGB3 signaling activation is involved in podocyte injury of SP^+^ mice.

### miR-30 knockdown induced uPAR-ITGB3 signaling through calcineurin-NFATC pathway in SP^+^ mice

**Fig. 2:**
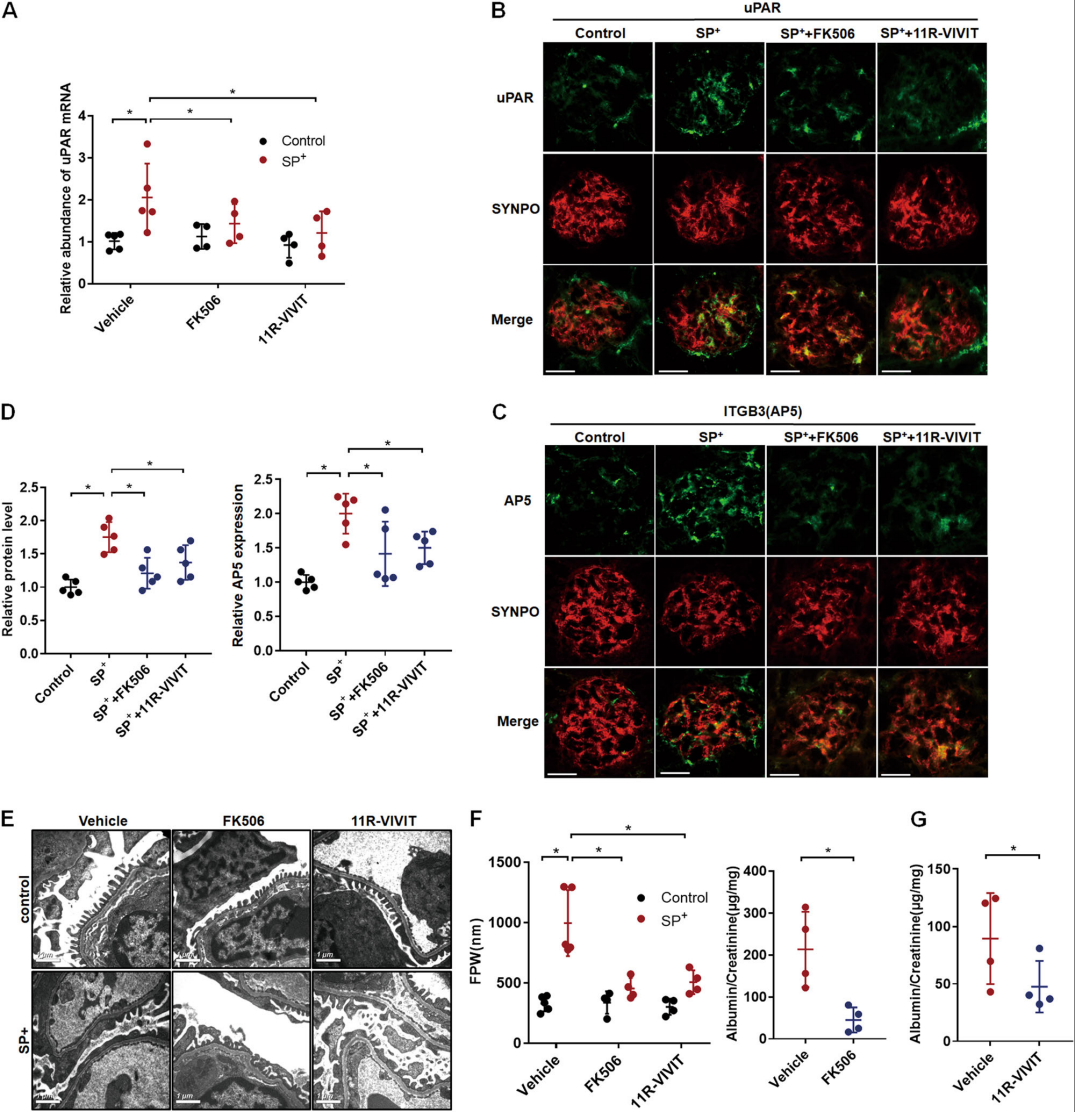
miR-30 sponge-induced uPAR-ITGB3 activation and podocyte injury were blocked by FK506 and 11R-VIVIT. **a** qRT-PCR analysis of uPAR in the isolated glomeruli from the SP^+^ mice that were untreated or treated with FK506 or 11R-VIVIT, indicating that miR-30 sponge-induced upregulation of uPAR mRNA can be inhibited by FK506 or 11R-VIVIT. Two-way ANOVA, *P < 0.05. **b**, **c** IF staining of uPAR, active ITGB3 and synaptopodin in podocytes of the SP^+^ mice untreated or treated with FK506 and 11R-VIVIT. Magnification in ×600. **d** Quantifications of the results in (**b**, **c**), indicating that both FK506 and 11R-VIVIT attenuated uPAR-ITGB3 activity. Two-way ANOVA, *P < 0.05. **e** EM showed that foot process effacement was partially alleviated by FK506 and 11R-VIVIT in the SP^+^ mice. Two-way ANOVA, *P < 0.05. **f**, **g** FK506 and 11R-VIVIT both significantly reduced albuminuria in the SP^+^ mice. Two-tailed student’s *T* test, *P < 0.05. All values were expressed as the means ± SD. Scale bar = 20 μm. *N* = 5 in both the control and SP^+^ groups; *N* = 4 in both the SP^+^ + FK506 and SP^+^ + 11R-VIVIT groups

We wondered how miR-30 knockdown could induce uPAR-ITGB3 activation. It has been reported that podocyte-specific transgenic expression of a constitutively active NFATC member can promote uPAR transcription through direct binding of NFATC to uPAR promoter, resulting in activation of ITGB3 and podocyte injury^18^. Since we have previously demonstrated that miR-30 functions to inhibit calcineurin signaling pathway by targeting its key components, including NFATC3, and that miR-30 downregulation leads to calcineurin and NFATC activation^8^, we speculated that calcineurin-NFATC pathway may mediate miR-30 deficiency-induced uPAR-ITGB3 signaling activation. We thus treated SP^+^ mice with calcineurin inhibitor, FK506, and NFATC inhibitor, 11R-VIVIT, respectively, and found significantly reduced expression of uPAR in podocytes of the SP^+^ mice at both mRNA and protein levels (Fig. 2a, b, d). FK506 and 11R-VIVIT also abrogated AP5 expression as shown by AP5 staining (Fig. 2c, d). Importantly, FK506 and 11R-VIVIT did partially attenuate podocyte foot process effacement (Fig. 2e) and albuminuria in the mice (Fig. 2f, g). Based on these results, it could be concluded that miR-30 inhibits uPAR-ITGB3 signaling through the calcineurin-NFATC pathway.

### In vitro, miR-30 knockdown also induced uPAR-ITGB3 activation and podocyte injury through calcineurin-NFATC pathway in cultured podocytes

**Fig. 3:**
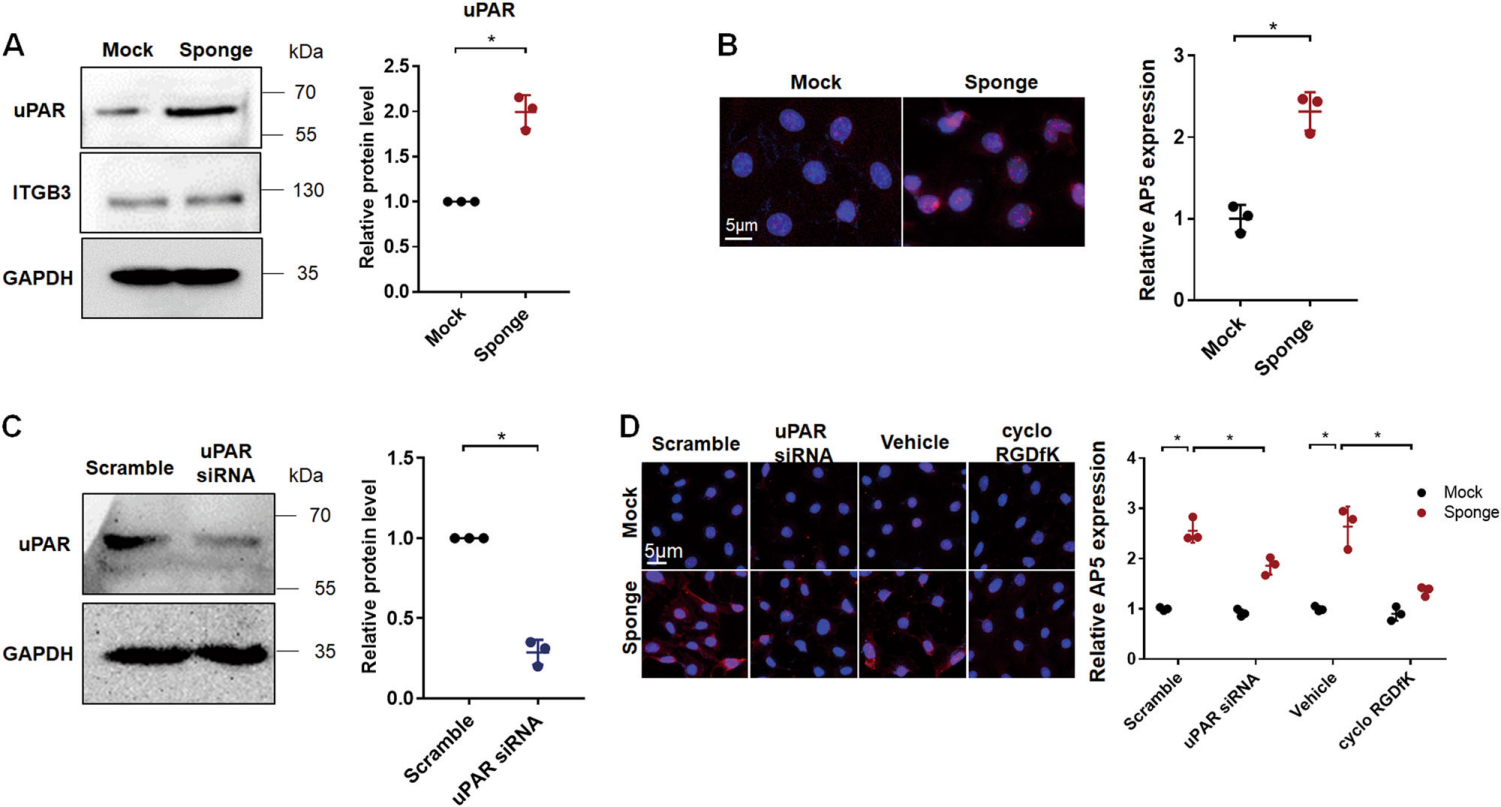
miR-30 knockdown induced uPAR-ITGB3 activation in cultured podocytes. **a** Western blotting showing the protein upregulation of uPAR by miR-30 sponge and the unchange of ITGB3. Quantification of the result was on the right based on three independent experiments. Two-tailed student’s *T* test, *P < 0.05 vs. mock group. **b** AP5 assay revealed that ITGB3 was prominently activated by miR-30 sponge (Magnification in ×600). Quantification of the results was on the right based on three independent experiments: two-tailed student’s *T* test, *P < 0.05. **c** Transfection of uPAR siRNA effectively reduced uPAR protein in the cells. Quantification of the result: Two-tailed student’s *T* test, *P < 0.05 vs. scramble group. **d** AP5 staining that detected ITGB3 activity demonstrated that miR-30 sponge-induced ITGB3 activation was blocked by uPAR siRNA and the ITGB3 inhibitor, cyclo-RGDfK (Magnification in ×600). Quantification of the results was shown on the right: Two-way ANOVA, *P < 0.05. All values were expressed as the means ± SD

We generated in vitro model of miR-30 knockdown-induced activation of uPAR-ITGB3 signaling by transfecting cultured podocytes with miR-30 sponge plasmid. We found, as expected, that uPAR expression was increased (Fig. 3a); meanwhile, although ITGB3 expression was not affected (Fig. 3a), its activity was significantly increased (Fig. 3b). To confirm that ITGB3 activation was caused by uPAR upregulation, we knocked down uPAR using siRNA (Fig. 3c) and observed significant attenuation of ITGB3 activation in the podocytes transfected with miR-30 sponge (Fig. 3d).

To confirm that uPAR-ITGB3 activation mediates miR-30 sponge-induced podocyte injury in vitro, we knocked down uPAR by siRNA or abrogated ITGB3 activation by cyclo-RGDfK in cultured podocytes that were treated with miR-30 sponge. We found that podocyte cytoskeletal injury was alleviated according to F-actin staining with phalloidin (Supplementary Fig. 1). As cytoskeletal rearrangement is involved in cell migration, scratch wound healing assay is routinely used to determine the occurrence of podocyte cytoskeletal change/injury under various stimuli. We therefore performed wound healing assay (Supplementary Fig. 2A, B), as well as the Real-time Cell Analysis that also measures cell motility (Supplementary Fig. 2C), and obtained results that were consistent with that of F-actin phalloidin staining.

**Fig. 4:**
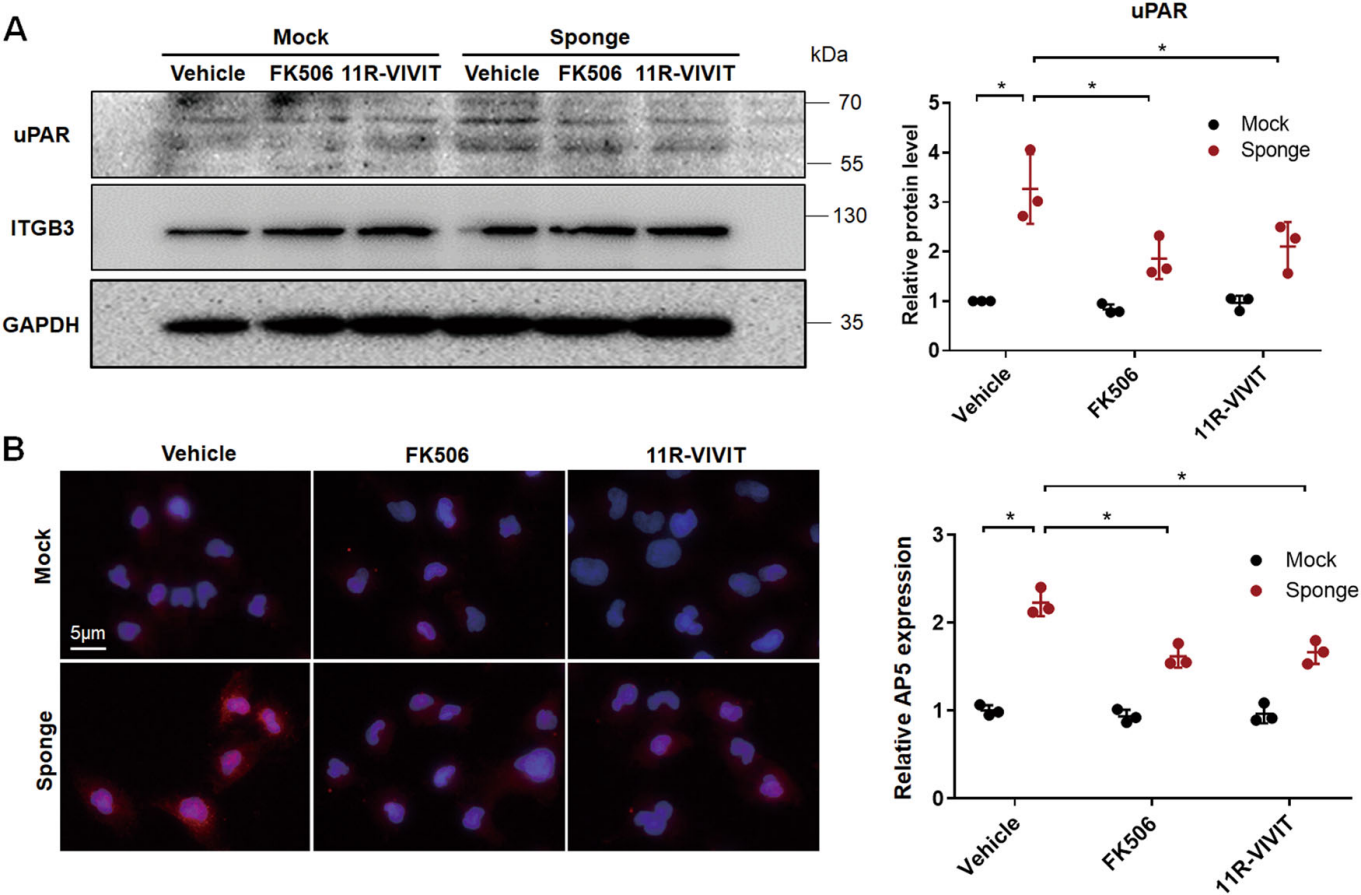
miR-30 regulated uPAR-ITGB3 signaling through calcineurin-NFATC in cultured podocytes. **a** Immunoblotting showed that uPAR was upregulated in the cells transfected with miR-30 sponge and that uPAR upregulation was attenuated by FK506 or the NFATC inhibitor, 11R-VIVIT. Of note, the ITGB3 protein level was not affected in the treatments. Quantification of the results was based on three independent experiments: Two-way ANOVA, *P < 0.05. **b** IF staining of AP5 showing that miR-30 knockdown activated ITGB3 in the podocytes. This effect of miR-30 sponge was diminished in the presence of FK506 or 11R-VIVIT (Magnification in ×600). Quantification of the results was on the right: Two-way ANOVA, *P < 0.05. All values were expressed as the means ± SD

We also found that FK506 and 11R-VIVIT similarly inhibited miR-30 sponge–induced uPAR-ITGB3 signaling in cultured podocytes, as they did in mice, including prevention of uPAR upregulation (Fig. 4a) and ITGB3 activation (Fig. 4b).

These results clearly demonstrated that miR-30 knockdown in cultured podocytes is a good in vitro model that mimics SP^+^ mice concerning the regulation of uPAR-ITGB3 signaling by miR-30.

### miR-30 deficiency-induced uPAR-ITGB3 activation led to Rho GTPases activation and synaptopodin downregulation in cultured podocytes

**Fig. 5:**
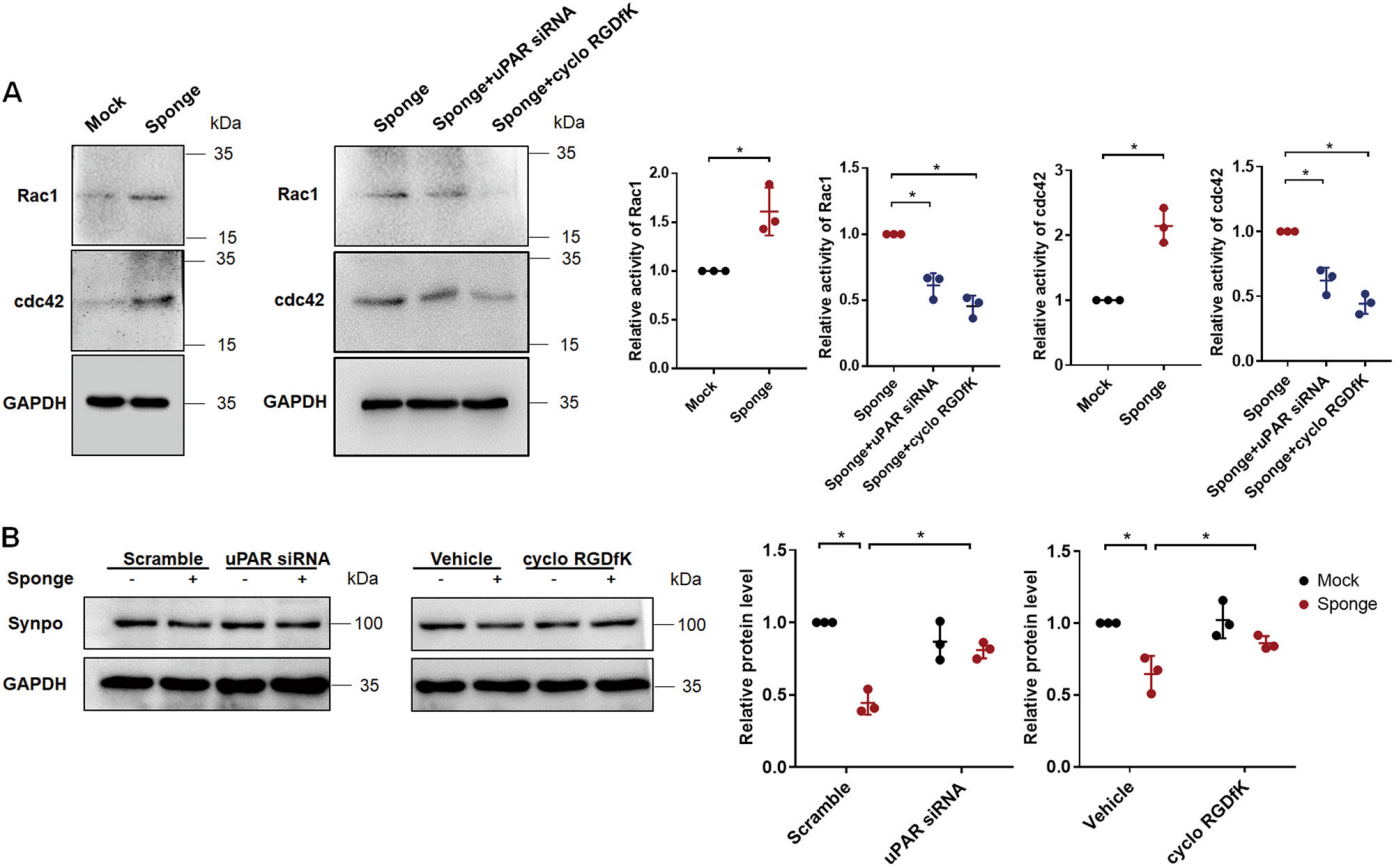
miR-30 sponge-induced uPAR-ITGB3 activation resulted in increases of RAC1 and CDC42 activities, and downregulation of synaptopodin. **a** Pull-down assay of active Rho GTPases showed that the activities of both RAC1 and CDC42 were increased by miR-30 sponge, but the increases were blocked by uPAR siRNA and cyclo-RGDfK. Quantifications of the results were shown based on three independent experiments: One-way ANOVA, *P < 0.05 verses mock group, or vs. the sponge + vehicle group, respectively. **b** Western blotting showing that both uPAR siRNA and cyclo-RGDfK reversed the miR-30 sponge-induced synaptopodin downregulation. Quantifications of the results based on three independent experiments: Two-way ANOVA, *P < 0.05. All values were expressed as the means ± SD

To explore how miR-30 deficiency-induced uPAR-ITGB3 activation causes podocyte injury, we examined the activity of Rho GTPases, RAC1 and CDC42, in cultured podocytes that were treated with miR-30 sponge. RAC1 and CDC42 are critical for podocyte cytoskeletal stability and their abnormalities in activity cause disruption of podocyte cytoskeleton^19^. It has previously been shown that ITGB3 activation alters RAC1 and CDC42 activities in podocytes^17^. We found that miR-30 knockdown by sponge increased the activities of both RAC1 and CDC42 and that this effect of miR-30 sponge was blocked by uPAR silencing and ITGB3 inhibition by cyclo-RGDfK (Fig. 5a), suggesting that uPAR-ITGB3 signaling may cause podocyte injury through altering RAC1 and CDC42 activities. We also examined the expression of synaptopodin, which is known to be essential for podocyte cytoskeleton, and found that synaptopodin expression was downregulated by the miR-30 sponge and restored by both uPAR siRNA and the ITGB3 inhibitor (Fig. 5b).

### miR-30 was downregulated in podocytes of LPS-treated mice, resulting in uPAR-ITGB3 activation and podocyte injury that were prevented by transgenic miR-30 expression

**Fig. 6:**
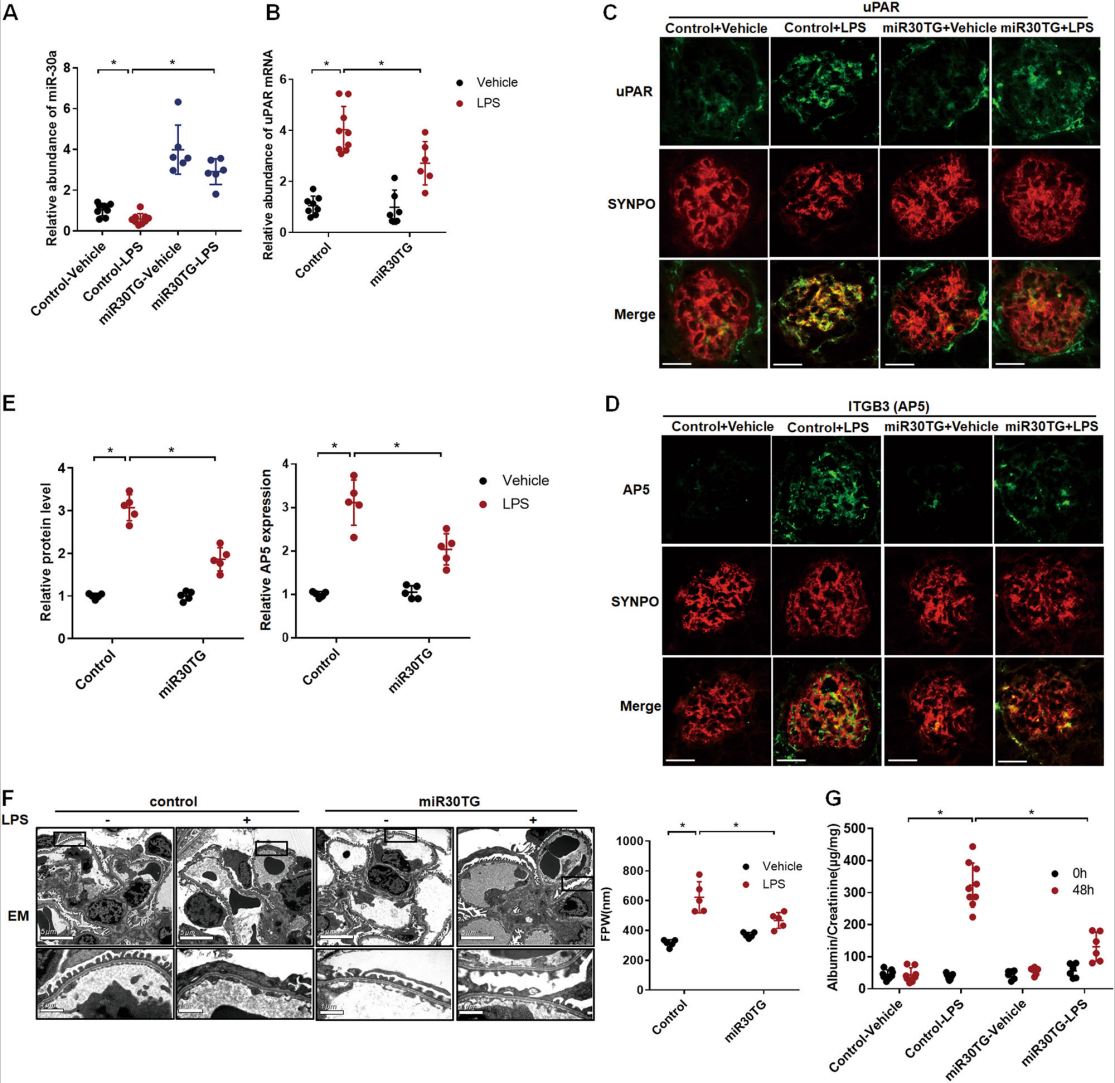
miR-30 downregulation in podocytes of LPS-treated mice resulted in uPAR-ITGB3 signaling activation and podocyte injury, which were prevented by transgenic miR-30acd expression. **a** qPCR analysis of miR-30a in glomeruli of the mice that were treated with LPS, showing downregulation of miR-30a vs. controls. One-way ANOVA, *P < 0.05. **b** qRT-PCR of uPAR in the glomeruli isolated from each group of mice showed that LPS increased uPAR mRNA but this effect was attenuated by transgenic miR-30acd expression (miR30TG). Two-way ANOVA, *P < 0.05. **c**, **d** Co-IF staining of uPAR (**c**) or AP5 (**d**) with synaptopodin confirmed the upregulation of uPAR protein and activation of ITGB3 in podocytes of the LPS-treated mice, and their attenuations by miR30TG in the podocytes (Magnification in ×600). **e** Quantifications of the results in (**c**, **d**): Two-way ANOVA, *P < 0.05. **f** Electron microscopic images showing severe foot process effacement in the LPS-treated mice. The severity of FPE ameliorated by miR30TG in the podocytes. Quantification of the foot process width (FPW) was shown on the right. Two-way ANOVA, *P < 0.05. **g** LPS induced significant albuminuria in mice, while miR30TG in podocytes significantly reduced albuminuria in the LPS-treated mice. Two-way ANOVA, *P < 0.05. All values are expressed as the means ± SD. Scale bar = 20 μm. *N* = 8 in the control + vehicle group, *N* = 9 in the control + LPS group, and *N* = 6 in both miR30TG + vehicle and miR30TG + LPS groups

To investigate the relevance of miR-30 deficiency-induced uPAR-ITGB3 activation with podocytopathy development, we used LPS-treated mice as a podocyte injury model. We found that LPS treatment resulted in downregulation of miR-30a expression in glomeruli of the mice (Fig. 6a), accompanied by uPAR upregulation (Fig. 6b, c and e) and ITGB3 activation (Fig. 6d, e). LPS treatment also resulted in podocyte foot process effacement and proteinuria, indicating that the podocyte injury model was successful.

To prove that downregulation of miR-30 was responsible for the LPS-induced uPAR-ITGB3 activation and podocyte injury, we generated conditional miR-30acd transgenic mice (CAG-loxP-RFP-stop-loxP-CFP-miR-30acd), which expressed exogenous miR-30a, -30c and -30d specifically in podocytes in the presence of NPHS2-Cre transgene (Supplementary Fig. 3). We chose miR-30a, -30c and -30d for transgenic expression in podocytes because they are the family members that are enriched in podocytes and downregulated in podocyte injury^7,8,20^. The transgenic miR-30acd expression was used to compensate endogenous miR-30 downregulation in podocytes of the LPS-treated mice such that the role for miR-30 downregulation in LPS-induced podocyte injury could be assessed. We found that double transgenic mice (CAG-loxP-RFP-stop-loxP-CFP-miR-30acd; NPHS2-Cre) exhibited sustained miR-30 expression under LPS treatment as shown by qPCR analysis of miR-30a in the glomeruli isolated from the LPS-treated double transgenic mice (Fig. 6a). The double transgenic mice also exhibited significant attenuation of uPAR upregulation and ITGB3 activation (Fig. 6b-e), as well as alleviation of foot process effacement and proteinuria (Fig. 6f, g). These results demonstrated that miR-30 downregulation was responsible for uPAR-ITGB3 activation and podocyte injury induced by LPS.

### LPS induced uPAR-ITGB3 activation and podocyte injury through downregulating miR-30 in cultured podocytes

**Fig. 7:**
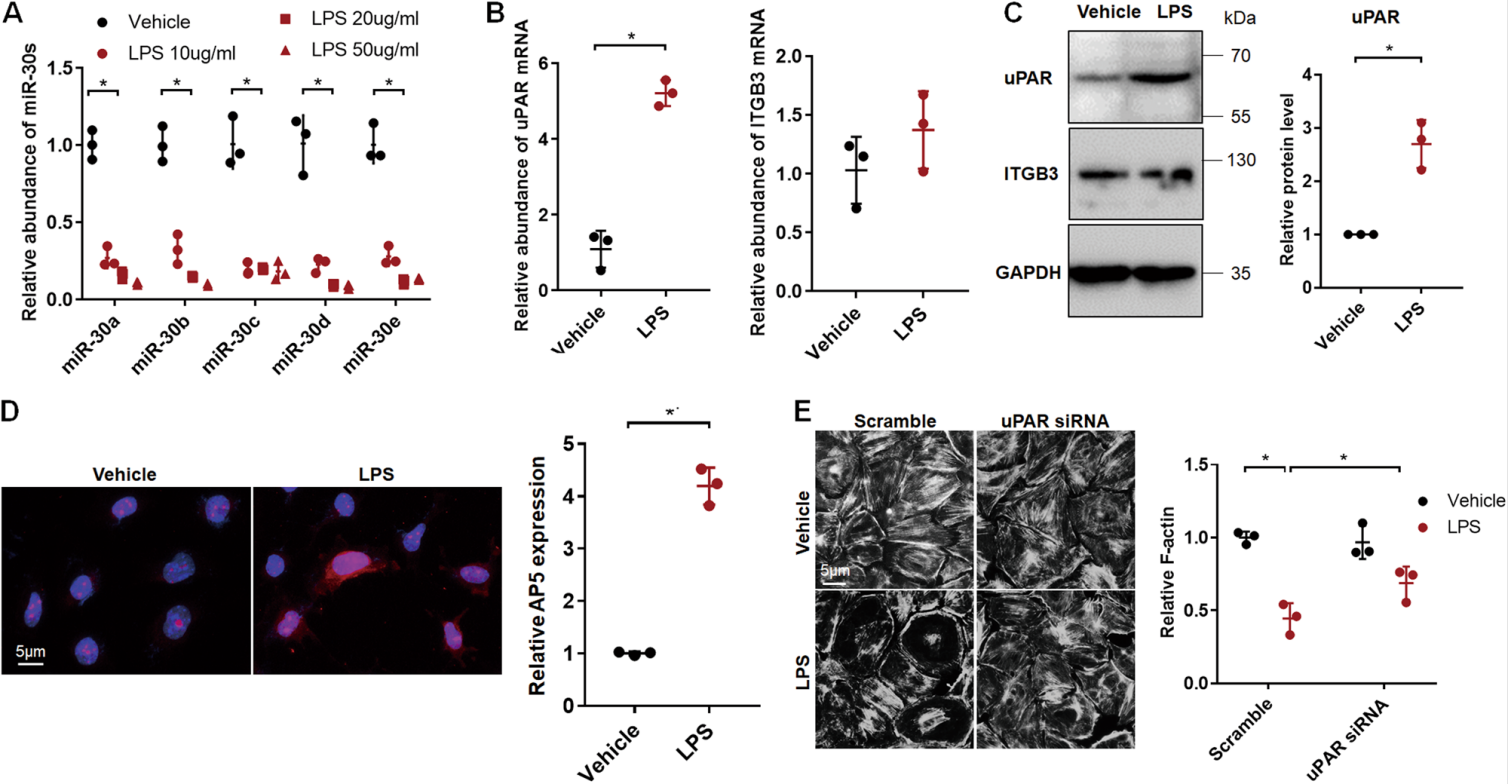
miR-30 downregulation in the LPS-treated podocytes in culture resulted in uPAR-ITGB3 activation and podocyte injury. **a** qRT-PCR showing that miR-30a through -30e were downregulated by LPS in a dose-dependent manner. One way ANOVA, *P < 0.05 vs. vehicle controls. **b** LPS increased mRNA expression of uPAR, but did not affect ITGB3 expression essentially. Two-tailed student’s *T* test, *P < 0.05 vs. vehicle controls. ITGB3 mRNA was not significantly increased in LPS treatment. **c** Western blotting of uPAR and ITGB3 in LPS-treated podocytes, showing that LPS upregulated protein of uPAR but not ITGB3. Quantification of the results based on three independent experiments: Two-tailed student’s *T* test, *P < 0.05 vs. vehicle controls. **d** IF AP5 staining showed that ITGB3 activity was increased by LPS (Magnification in ×600). Quantification of the results: two-tailed student’s T test, *P < 0.05 vs. vehicle control. **e** Phalloidin staining showing the loss of F-actin stress fibers in LPS-treated podocytes and the restoration by uPAR knockdown (Magnification in ×600). Quantification of the results: Two-way ANOVA, *P < 0.05 vs. vehicle + scramble control. Cells were treated with 20 μg/ml LPS or vehicle for 24 h before harvest. All values were expressed as the means ± SD

We determined the effect of LPS on miR-30 expression in cultured podocytes and found that LPS greatly downregulated all miR-30 family members (Fig. 7a), accompanied by uPAR upregulation (Fig. 7b, c) and ITGB3 activation (Fig. 7d), increase of RAC1 and CDC42 expression (Supplementary Fig. 4), as well as cytoskeletal injury. uPAR siRNA alleviated LPS-induced cytoskeletal injury (Fig. 7e), indicating that uPAR-ITGB3 signaling mediated the LPS-induced podocyte injury.

To confirm the effect of uPAR on cultured podocytes, we overexpressed uPAR by transfecting pCMV3-HA-uPAR plasmid into podocytes (Supplementary Fig. 5A) and found that ITGB3 was activated (Supplementary Fig. 5B) and cell motility was enhanced, indicating cytoskeletal alteration in the podocytes (Supplementary Fig. 5C).

**Fig. 8:**
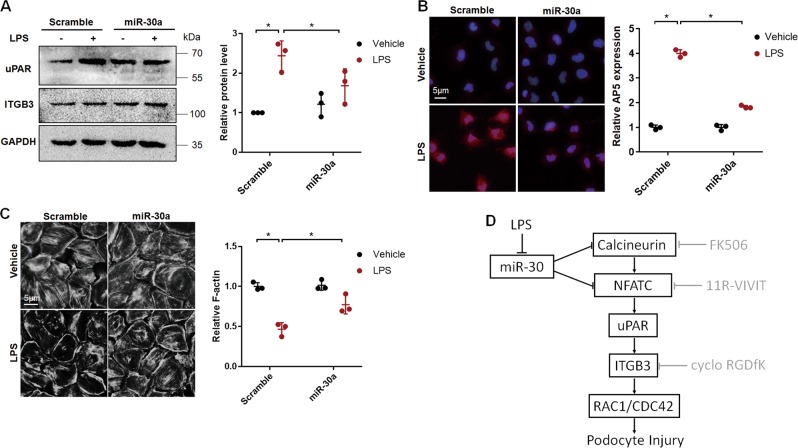
miR-30a overexpression ameliorated uPAR-ITGB3 activation and podocyte injury in cultured podocytes. **a** Western blotting of uPAR and ITGB3 revealed that uPAR was upregulated by LPS, while exogenous miR-30a transfection prevented LPS-induced uPAR upregulation; in contrast, ITGB3 expression was not affected in the treatments. Quantification based on three independent experiments was shown on the right. **b** IF staining of AP5 indicated that miR-30a mimic inhibited LPS-induced ITGB3 activation (Magnification in ×600). (**c**) Phalloidin staining showing that LPS induced F-actin fiber loss in the cells, which it could be ameliorated by miR-30a. The quantifications of the results in (**a**–**c**) were each on the right, and Two-way ANOVA was used for the comparison with *P < 0.05 considered statistically significant. Cells were treated with 20 μg/ml LPS or vehicle for 24 h before harvest. All values were expressed as the means ± SD. **d** The relationship among miR-30, uPAR-ITGB3 pathway, calcineurin-NFATC pathway, and the actions of FK506, 11R-VIVIT and cyclo-RGDfK

Similarly, when we transfected podocytes with miR-30a mimic to compensate LPS-induced miR-30 loss, we found that uPAR upregulation (Fig. 8a), ITGB3 activation (Fig. 8b), and cytoskeletal injury (Fig. 8c) were all attenuated in the cells treated with LPS, indicating that miR-30 downregulation was responsible for uPAR upregulation, ITGB3 activation, and podocyte injury.

## Discussion

We have previously shown that miR-30 family is essential for podocyte homeostasis and its downregulation causes activation of signaling pathways that lead to podocyte injury, eg., calcium/calcineurin^8^. Restoration of miR-30 expression has been found to underlie the therapeutic effect of glucocorticoids on podocytopathies^7^. Therefore, miR-30 is central to podocyte homeostasis and injury. In recent years, uPAR has been shown to be upregulated in podocytes in multiple glomerular diseases and trigger ITGB3 signaling that leads to podocyte injury^17^. These studies motivated us to investigate the relationship between miR-30 and uPAR-ITGB3 signaling. Our previous preliminary study has shown that miR-30 knockdown is capable of inducing uPAR-ITGB3 activation in cultured podocytes^8^. In the present study, we performed extensive studies using several in vivo and in vitro models and demonstrated that miR-30 family normally acts to prevent uPAR expression in podocytes and that when miR-30 downregulation occurs, uPAR is upregulated and activates ITGB3 signaling, leading to Rho GTPase activity alterations and podocyte injury. Furthermore, we show that miR-30 family inhibits uPAR-ITGB3 signaling through inhibiting the calcineurin-NFATC pathway. Therefore, our studies have uncovered the interactions between the crucial players in podocyte injury. The relationships among miR-30, uPAR-ITGB3 pathway, calcineurin-NFATC pathway and their inhibitors are summarized according to the study. (Fig. 8d)

As a common hallmark of most proteinuric kidney diseases, podocyte foot process effacement (FPE) occurs through cytoskeletal reorganization^21^. FPE acts as a protective response to local injurious stimuli, resulting in formation of tight junctions that prevent podocyte detachment^22^. On the other hand, FPE is detrimental because it causes filtration barrier damage and proteinuria^23^. In the present study, we have shown that podocyte-specific miR-30 sponge transgenic mice develop FPE and proteinuria, which are accompanied by activation of uPAR-ITGB3 signaling, and that uPAR-ITGB3 activation in turn induces podocyte injuries as evidenced by amelioration of FPE and proteinuria by the ITGB3 inhibitor.

We further explored the events downstream uPAR-ITGB3 signaling and found that RAC1 and CDC42 were increased in activity by uPAR-ITGB3 signaling as both uPAR silencing and ITGB3 inhibition prevented miR-30 sponge-induced upregulation of RAC1 and CDC42 activities. RAC1 and CDC42 are two of the Rho GTPases family members that play critical roles in podocyte cytoskeletal dynamics and stability. Alterations of their activities, either increase or decrease, cause podocyte cytoskeletal injury that leads to podocyte structural change, filtration barrier disruption and proteinuria^19,24^. Consistently, earlier studies have indeed shown that functional inhibition of uPAR or ITGB3 can prevent LPS-induced activity increase of RAC1 and CDC42 in podocyte^17^. Together, these studies suggest that miR-30 deficiency-induced uPAR-ITGB3 signaling activation causes podocyte injury through altering RAC1 or CDC42 activity. Nevertheless, it remains unknown how uPAR-ITGB3 signaling affects RAC1 and CDC42 activities and warrants further investigations.

We also investigated how miR-30 downregulation causes uPAR-ITGB3 signaling activation and speculated that it might involve calcineurin-NFATC pathway. We have previously shown that miR-30 normally functions to suppress calcium/calcineurin activation^8^ which is known to induce cytoskeletal injury and apoptosis in podocytes through multiple pathways^25^, including NFATC^10,26^. NFATC family members are the substrates of calcineurin, and upon dephosphorylation by calcineurin they are activated and translocated to nuclei where they function as transcriptional factors to promote target gene expression. NFATC activation in podocytes causes progressive proteinuria and glomerular sclerosis in mice^27^, and the NFATC inhibitor is capable of ameliorating proteinuria and podocyte injury in diabetic mice^28^. Importantly, it has been recently shown that NFATC can promote uPAR expression to induce podocyte injury^18^. These studies suggest that miR-30 deficiency induces activation of calcineurin-NFATC pathway, which, in turn, directly upregulates uPAR expression at transcriptional level in podocytes. Supportively, we found in this study that both calcineurin inhibitor (FK506) and NFAT inhibitor (11R-VIVIT) reversed miR-30 sponge-induced upregulation of uPAR, as well as activation of ITGB3, accompanied by significant amelioration of podocyte foot process effacement and proteinuria in podocyte-specific miR-30 sponge transgenic mice (Fig. 2). Similar results were obtained from studies using cultured podocytes in vitro (Fig. 4).

Having clearly demonstrated the interactions between miR-30, calcineurin-NFATC and uPAR-ITGB3 using miR-30 sponge knockdown in podocytes in vivo and in vitro, we have further demonstrated their regulatory relationship in podocyte injury model of LPS-treated mice and cultured podocytes, and have thus shown a clinical relevance of our present study. We showed that LPS induced miR-30 downregulation, uPAR upregulation, and ITGB3 activation, accompanied by podocyte injury in mice or cultured podocytes. These effects of LPS were abrogated by the exogenous miR-30 expression, uPAR siRNA, ITGB3 inhibitor, as well as FK506 and 11R-VIVIT. These results demonstrate that miR-30 deficiency-induced calcineurin-NFATC activation followed by uPAR-ITGB3 signaling activation underlies LPS-induced podocyte injury and that sustaining miR-30 expression is sufficient to alleviate the injurious effects of LPS on podocytes.

Apart from miR-30 downregulation and uPAR signaling activation, multiple other mechanisms by which LPS induces podocyte injury have been reported previously. For example, LPS can activate MAPK p38^29^ and NF-κB by acting on TLR4^30^. In the present study, we focused only on the miR-30-calcuneurin/NFAT-uPAR/ITGB3 pathway and have shown its contribution to LPS-induced podocyte injury. It is necessary to determine whether these pathways act independently or synergistically in LPS-induced podocyte injury in the future.

Together with the earlier studies that have shown miR-30 inhibits multiple injurious pathways, including TGF-β^20^, notch^7^, calcium/calcineurin^8^ and angiotensin^31^, in podocytes, our present study suggests that miR-30 family might be an ideal target for therapeutic intervention of various podocytopathies.

In addition to ITGB3, uPAR has also been reported to interact with other integrins, eg., ITGB2, on the surface of neutrophils and monocytes to affect the migration and tissue recruitment of these cells^32^. Podocytes are known to attach to GBM via integrin β1. However, under injurious stimulations, uPAR, uPA and PAI are ligated to induce endocytosis and loss of integrin β1 on the cell surface, leading to podocyte detachment^33^. This could be an alternative mechanism by which uPAR upregulation causes podocyte injury and loss in our experimental system. More recently, uPAR has been found to promote AKT2 phosphorylation, leading to α_M_β2 integrin upregulation, intracellular calcium increase and neutrophil migration enhancement^34^. In the present study, we have only focused on uPAR-ITGB3 pathway, however, other pathways, such as those mentioned above, could also be involved in the podocyte injury.

To assess how common uPAR-ITGB3 signaling activation is in podocytes in glomerular diseases, we searched the database of Nephroseq and found that uPAR is also upregulated in the glomeruli of diabetic nephropathy (Supplementary Fig. 6A), vasculitis (Supplementary Fig. 6B) and lupus nephritis (Supplementary Fig. 6C). It is highly likely that the upregulations of uPAR in podocytes of these diseases are also induced by miR-30 downregulation and facilitate podocyte injury. Further investigation is required to confirm this speculation.

Altogether, our present study has proven that miR-30 family suppresses uPAR-ITGB3 signaling in mice and cultured podocytes through inhibiting activation of calcineurin/NFATC pathway; and that miR-30 downregulation leads to podocyte injury through calcineurin-NFATC activation followed by uPAR-ITGB3 activation. This mechanism underlies podocyte injury induced by LPS as shown in the present study. It may also underlie podocyte injury in other glomerular diseases. Besides, this study has also elucidated the relationship among the crucial players governing podocyte pathophysiology and the actions of the drugs commonly used for the treatment of podocytopathies, and thus underscored the therapeutic intervention of podocytopathies based on miR-30.

## Materials and Methods

### Podocyte culture, treatment and transfection

The immortalized human podocytes were provided by Dr. Saleem M (University of Bristol, United Kingdom) and were cultured as previously described^35^. The podocytes were treated as follow: 10 μg/ml, 20 μg/ml, or 50 μg/ml LPS (L-2880, Sigma Aldrich, MO, USA); 1 μM FK506; 100 nM 11R-VIVIT (480401, Merck, Germany); 1 μM cyclo-RGDfK (S7834, Selleck, USA). The detailed treatments and their combinations are indicated in the text. For transient transfection of plasmid DNA or siRNA, Lipofectamine 2000 (Invitrogen, USA) was used according to the manual instructions.

### RNA extraction and qRT-PCR

Total RNA was extracted using Trizol Reagent (Invitrogen, USA) according to the manufacturer’s instructions. MicroRNA molecules were reverse transcribed with AMV (Takara, Japan) and quantified using TaqMan miRNA Assay Probes (Applied Biosystems, Foster City, CA). mRNA was reverse transcribed using the One-Step PrimeScript RT-PCR Kit (Takara, Japan) and quantified using SYBR Premix Ex Taq (Takara, Japan). qRT-PCR was performed with the 7900HT Sequence Detection System (Applied Biosystems). miRNA expressions were normalized to U6 snRNA. mRNA levels were normalized to GAPDH. Primers were synthesized by Tsingke Biological Technology (Nanjing, China). Primer sequences are listed blow.

#### Primer sequences used in this study for qPCR

**Table:** 

Genes	Forward (5′–3′)	Reverse (5′–3′)
h-uPAR	CCCCATGAATCAATGTCTGGT	ACAGCCACTTTTAGTACAGCA
h-ITGB3	ACATGACGAAAATACCTGCAACC	GGCTCTTCTACCACATACAGGA
h-GAPDH	TCTGACTTCAACAGCGACACC	GTTGCTGTAGCCAAATTCGTT
m-uPAR	ACAGCAGGTTTCCATAGCAA	TGTAACACTGGAAGCCATTCG
m-ITGB3	TGATGACTCAAGCAACGTCCT	CGATACTAA AGCTCACCGTGTC
m-GAPDH	CCATCTTCCAGGAGCGAGAC	TTTCTCGTGGTTCACACCCAT

### Western blotting

Cultured podocytes were lysed in RIPA buffer containing protease inhibitor cocktail and phosphatase inhibitors (Roche). The lysates were fractionated in SDS-PAGE, followed by protein transfer to blots and incubation with primary antibodies, including polyclonal anti-uPAR (1:200, SC-10815, Santa Cruz, Texas, USA), polyclonal anti-ITGB3 (1:200, SC-14009, Santa Cruz, Texas, USA), polyclonal anti-synaptopodin (1:200, SC-21536, Santa Cruz, Texas, USA), and polyclonal anti-GAPDH (1:5000, AP0063, Bioworld, MN, USA), respectively.

### Wound-scratch assay

Podocytes were cultured in 6-well plates (Corning, NY, USA). Podocytes were starved in RPMI1640 for 16 h before transfection. Cells were transfected with vector control plasmid, miR-30 sponge plasmid, scramble siRNA, and uPAR siRNA, separately or in combinations, followed by treatment with cyclo-RGDfK or vehicle control as indicated in the main text, according to the manufacturer’s instructions. The cells scratches were made using a sterile pipette tip, followed by imaging under an inverted phase-contrast microscope (Nikon Corporation, Japan). Then podocytes were cultured in 37℃ and pictures were obtained 24 h later. The areas of scratches were quantified using imageJ (National Institutes of Health, Bethesda, MD) and the relative motility of the cells was calculated by the area in an initial scratch where they migrated and occupied in 24 h.

### Real-Time Cell Analysis (RTCA)

Podocyte mobility was measured using xCELLigence RTCA DP System (ACEA Bioscience Inc, CA, USA). Before the cells were seeded, the CIM-16 plate was balanced and baseline measurement was performed. Then cells were trypsinized and counted using the Scepter^TM^ Handheld Automated Cell Counter and Scepter^TM^ Sensors 60 μm (Millipore Corporation, Massachusetts, USA). These cells were then seeded in the upper chamber of a CIM-16 plate at a density of 8000 cells/well in RPMI medium with 10% FBS. The plate was placed in electronically integrated cradle. The impedance values (Cell Index, CI) of the membrane between the upper chamber and lower chamber was measured in real-time, which represented the quantitative movement of the cells through the membrane. The CI data were collected every 2 h and analyzed by the Real-Time Cell Analysis (RTCA) software 2.0.

### Actin cytoskeleton staining and quantification

The cultured podocytes were fixed with 4% paraformaldehyde at room temperature. The cells were permeabilized with 0.1 % Triton X-100 and then blocked with 10% FBS. Rhodamine-labeled phalloidin was used to stain F-actin, and the images were captured under a Leica microscope (DM5000B). The stained areas of actin fibers per cell were quantified with ImageJ software (National Institutes of Health, Bethesda, MD).

### Cell immunofluorescence staining

Cells were cultured in slide chamber (ThermoFisher, Massachusetts, USA). After the treatment, the cells were rinsed with cold PBS for 3 times, fixed with 4% paraformaldehyde, permeabilized with 0.1% triton, blocked with 10% FBS, and then incubated with monoclonal anti-ITGB3 (GPIIIa, CD61), PSI domain [AP-5] (1:100, P05106, Kerafast, USA). Then cells were incubated with Cy3-conjugulated anti-mouse antibody (1:200, A0521, Beyotime Biotechnology, Jiangsu, China). The nuclei were stained with DAPI. The slides were examined using a Leica microscope (DM5000B).

### Rho GTPase activation assay

We measured the GTPase activity of RAC1 and CDC42 using their activation detection biochemical kit (BK-034, BK-035, Cytoskeleton Inc. Denver, USA), respectively, following the manufacturer’s instruction. Cells were treated and lysed quickly, followed by snap freezing in liquid nitrogen.

### Animals

Transgenic mice specifically expressing a miR-30 sponge in podocytes were described previously^8^. 10 week -old SP^+^ and control mice were used in the studies. Podocyte-specific miR-30 transgenic mice (Pod-miR-30) were generated by the Nanjing Biological Research Institute of Nanjing University. In this Pod-miR-30 transgene, a CAG-LoxP-RFP-Stop-LoxP-CFP cassette preceded the sequence encoding mature miR-30a, miR-30c and miR-30d (Fig. 7). The transgene was identified using PCR genotyping with the primers as follows: Pod-miR-30-fwd, 5′-CCATCAGCGACAACGTCTATATCAC; Pod-miR-30-rev, 5′-TATTCGACCACATGGGCTGAGTAAG. This transgene was crossed to podocyte-specific *NPHS2-Cre* transgene to produce double transgenic mice for the experiments.

To induce podocyte injury in mice, 20 mg/kg LPS was injected intraperitoneally twice with an interval of 24 h. Twelve hours after each injection, the mice received warm normal saline i.v. to reduce LPS-induced sepsis responses. Urine and kidney samples were collected 48 h after the first injection for analysis. FK506 was injected i.p at 3 mg/kg daily. 11R-VIVIT was injected i.v at 1 mg/kg daily. Cyclo-RGDfK was injected i.v at 10 mg/kg every 8 h for a total of 3 times.

### Urinary albumin and creatinine measurements

Urinary albumin and creatinine levels of the mice were measured using Albuwell M (Exocell, Philadelphia, USA) and QuantiChrom™ Creatinine Assay Kit (Bioassay systems, CA, USA.) according to the manufacturer’s instructions. The results were presented as albumin/creatinine ratio (ACR, μg/mg).

### Transmission electron microscopy and quantification of podocyte foot process effacement

Renal cortex tissues were cut into 1-mm^3^ pieces, immediately fixed in 3.75% glutaraldehyde, and post-fixed in phosphate-buffered 1% osmium tetroxide. After dehydration, the specimens were embedded in epoxy resin. Ultrathin sections (70 nm) were stained and examined by the Hitachi 7500 transmission electron microscope (Hitachi, Tokyo, Japan). Electron microscopy images were analyzed with Gatan software. We quantified the podocyte foot-process effacement following the method described previously^36,37^.

### Immunofluorescence Staining

A 5-μm sections of mice frozen tissues were blocked with 10% FBS and incubated with primary antibodies, monoclonal anti-PLAUR/uPAR (1:100, SAB4200412, **S**igma Aldrich, MO, USA), monoclonal anti-ITGB3 (GPIIIa, CD61), PSI domain [AP-5] (1:100, P05106, Kerafast, USA), and polyclonal anti-synaptopodin (1:200, SC-50459, Santa Cruz, Texas, USA), respectively. The sections were then incubated with an FITC-conjugated anti-mouse secondary antibody (1:200, A0568, Beyotime, Jiangsu, China) or a Cy3-conjugated anti-rabbit antibody (1:200, A0516, Beyotime, Jiangsu, China). The images were captured under the Leica microscope (DM5000B). The results were quantified using 5 different images captured randomly in each group.

### Statistical Analyses

The data are presented as the mean ± SD. The results were analyzed using GraphPad Prism 6 software (GraphPad Software Inc., CA, USA). The differences between two groups were analyzed using a two-tailed Student’s *t* test. ANOVA was used for comparisons among multiple groups. Differences were considered statistically significant when the two-sided P value <0.05.

### Study approval

The animals and experimental procedures were approved by the Institutional Animal Care and Use Committee of Jinling Hospital, Nanjing University School of Medicine.

## Supplementary information

CDDIS-19-0692 Supplementary Material
