# Supplementary material for: MiR-30 family prevents uPAR-ITGB3 signaling activation through calcineurin-NFATC pathway to protect podocytes
Source: Cell Death Dis. 2019 May 24;10(6):401. doi: 10.1038/s41419-019-1625-y (PMC6534572; doi:10.1038/s41419-019-1625-y)
Supplement: Supplementary file 1 — CDDIS-19-0692 Supplementary Material [file 41419_2019_1625_MOESM1_ESM.pdf]

Supplementary Material

MiR-30 Family Prevents uPAR-ITGB3 Signaling Activation Through Calcineurin-NFATC Pathway to Protect Podocytes

Yue Lang[#1], Yue Zhao[#1], Chunxia Zheng[1], Yinghui Lu[1], Junnan Wu[1], Xiaodong Zhu[1], Mingchao Zhang[1], Fan Yang[1], Xiaodong Xu[1], Shaolin Shi[1*], Zhihong Liu[1*]

[1] National Clinical Research Center of Kidney Diseases, Jinling Hospital, Nanjing University School of Medicine, Nanjing, Jiangsu 210002, China

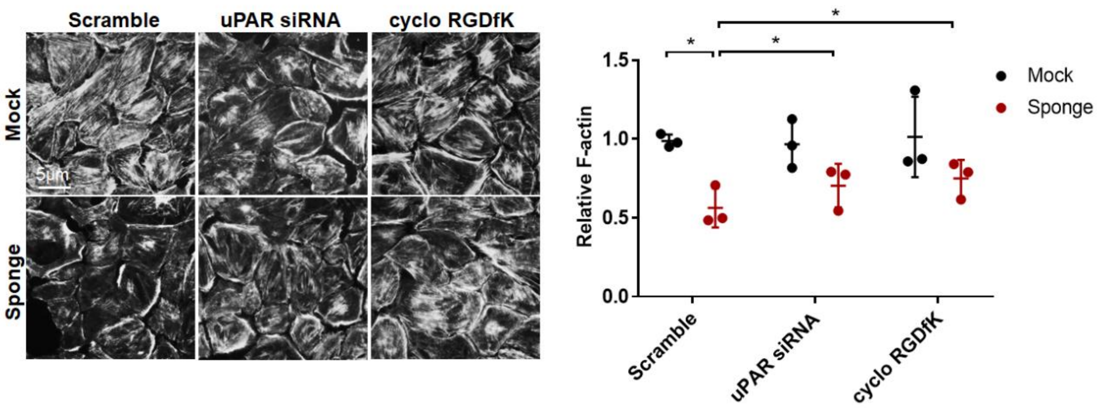

Supplementary Figure 1. uPAR-ITGB3 signaling mediated miR-30 sponge-induced cytoskeleton damage. Phalloidin staining of F-actin showed that miR-30 sponge caused loss of F-actin stress fibers in cultured podocytes, which was alleviated by uPAR siRNA and integrin β3 inhibitor. Quantifications of the results were performed and Two-way ANOVA was used for the comparisons, *P<0.05 considered statistically significant.

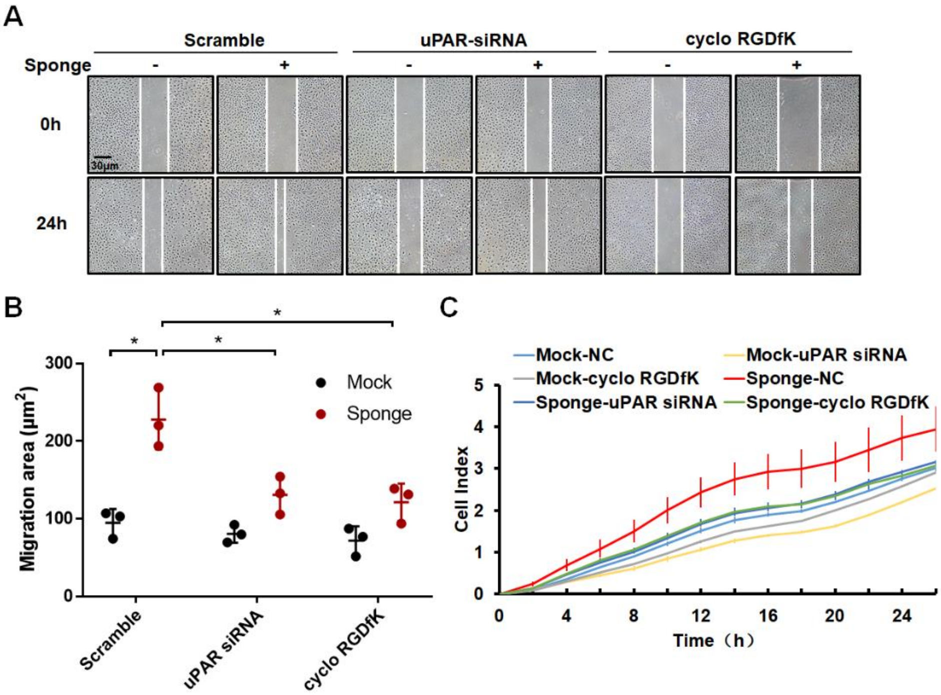

Supplementary Figure 2. uPAR-ITGB3 signaling mediated miR-30 sponge-induced motility enhancement of the cultured podocytes. (A) Wound healing assay showing enhanced cell motility in miR-30 sponge-treated podocytes, which was abolished by uPAR siRNA co-transfection or integrin β3 inhibitor (Magnification 100×). (B) Quantification of the results in A. Two-way ANOVA, *P<0.05. (C) The Real-Time Cell Analysis of podocytes consistently showing similar results as in the wound healing assay in B. n=4 in each group.

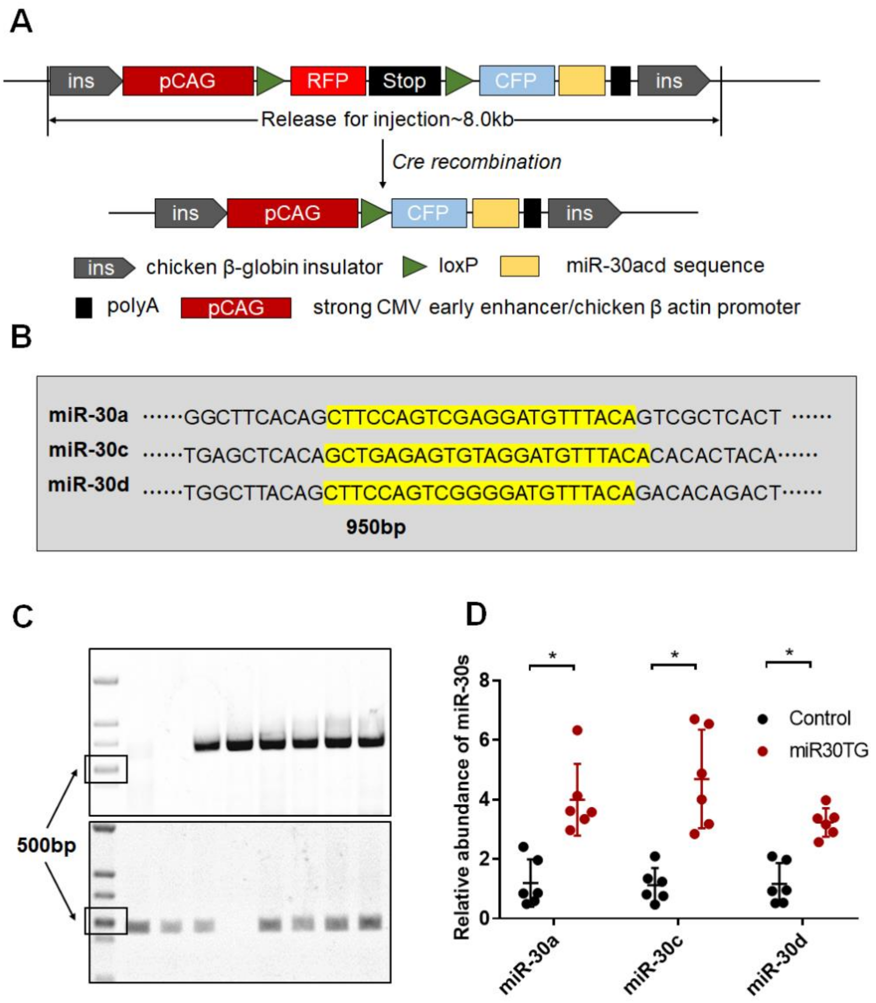

Supplementary Figure 3 The generation of miR-30acd transgenic mice (miR30TG). (A) Schematic representation of the generation of podocyte-specific miR30TG mice. (B) Sequence of the miR-30acd transgene. (C) PCR genotyping of miR30TG mice that gave rise to a 542 bp product, and the NPHS2-Cre transgenic mice that yielded a 481 bp product. (D) qPCR analysis of miR-30a, -30c and -30d in the glomeruli of podocyte-specific miR30TG mice and control mice, showing that miR-30a, -30c, and -30d were indeed increased in the transgenic mice. Two-tailed student's T test, *P<0.05 versus control mice.

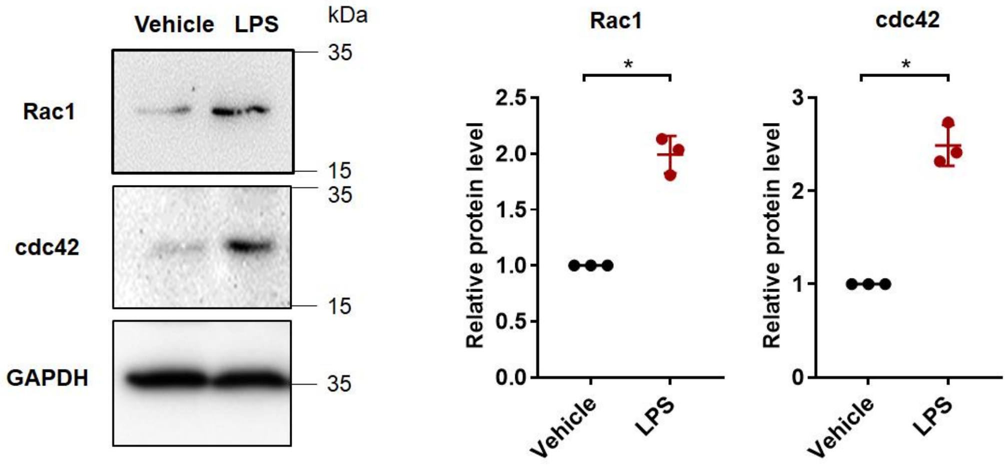

Supplementary Figure 4. LPS upregulated RAC1 and CDC42 expressions in cultured podocytes. Quantification of the results was shown on the right. Two-tailed student’s T test, *P<0.05 versus vehicle control.

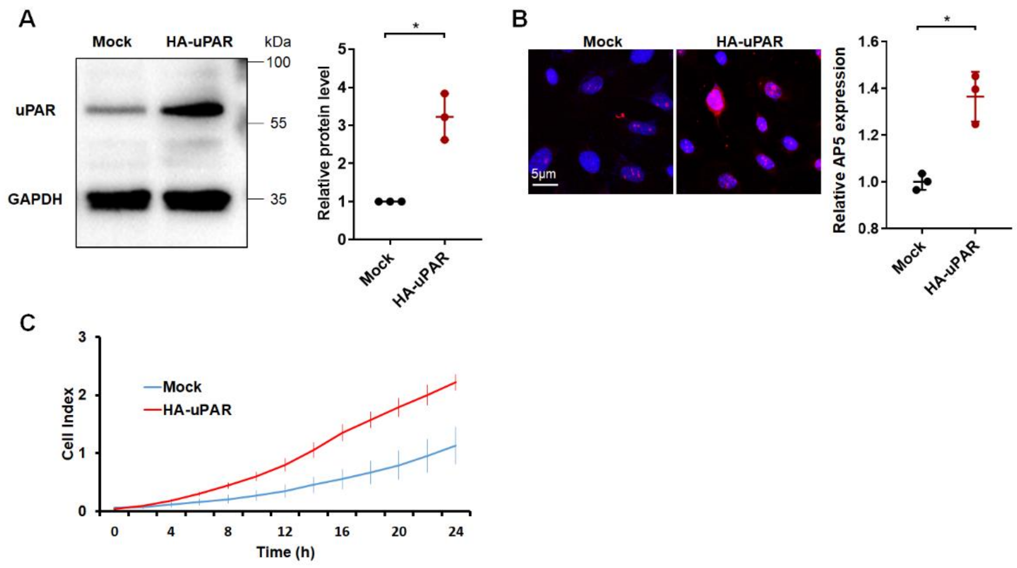

Supplementary Figure 5. uPAR overexpression activated ITGB3 and caused enhanced podocyte migration. (A) pCMV3-PLAUR-HA plasmid transfection increased uPAR protein in the podocytes. Two-tailed student’s T test, *P<0.05 versus mock control. (B) uPAR overexpression in podocytes activated ITGB3 (Magnification 600×). Quantification of the results was shown on the right. Two-tailed student’s T test, *P<0.05 versus mock control. (C) uPAR overexpression enhanced podocyte motility in the Real-time Cell Analysis. All values are expressed as the means±SD.

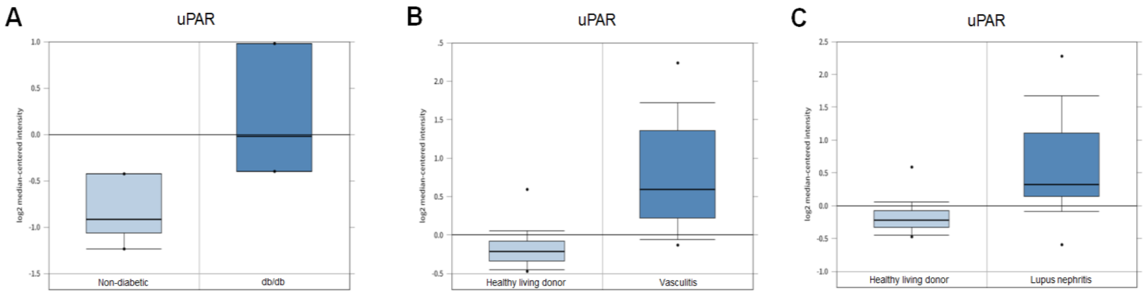

Supplementary Figure 6. uPAR is up-regulated in glomeruli of various kidney diseases according to Nephroseq (Hodgin Diabetes data). (A) uPAR expression is upregulated in Hodgin Diabetes data , Group: Diabetic db/db mice vs. Non-diabetic mice. (B) uPAR expression is upregulated in the glomeruli of patients with vasculitis compared with healthy living donors (Ju CKD Glomerular data). (C) uPAR is upregulated in glomeruli of the patients with lupus nephritis compared with healthy living donors (Ju CKD Glomerular data).
